# Treatment of xerostomia and hyposalivation in the elderly: A systematic review

**DOI:** 10.4317/medoral.20969

**Published:** 2016-03-31

**Authors:** José-Antonio Gil-Montoya, Francisco-Javier Silvestre, Rocío Barrios, Javier Silvestre-Rangil

**Affiliations:** 1 Instituto de Investigación Biosanitaria de Granada, School of Dentistry, University of Granada; 2 Stomatology Department, School of Medicine and Dentistry, University of Valencia

## Abstract

**Background:**

Therapeutic strategies for xerostomia, regardless of etiology, have so far not had definitive or clearly effective results. Objectives. To systematically revise the latest scientific evidence available regarding the treatment of dry mouth, regardless of the cause of the problem.

**Material and Methods:**

The literature search was conducted in March 2015, using the Medline and Embase databases. The “Clinical Trial”, from 2006 to March 2015, was carried out in English and only on human cases. The draft of the systematic review and assessment of the methodological quality of the trials was carried out following the criteria of PRISMA (Preferred Reporting Items for Systematic Reviews and Meta-Analyses) and the “Oxford Quality Scale”.

**Results:**

Finally, a total of 26 trials were identified that met the previously defined selection and quality criteria; 14 related to drug treatments for dry mouth, 10 with non-pharmacological treatment and 2 with alternative treatments.

**Conclusions:**

Pilocarpine continues to be the best performing sialogogue drug for subjects with xerostomia due to radiation on head and neck cancer or diseases such as Sjogren’s Syndrome. For patients with dry mouth caused solely by medication, there are some positive indications from the use of malic acid, along with other elements that counteract the harmful effect on dental enamel. In general, lubrication of oral mucous membrane reduces the symptoms, although the effects are short-lived.

**Key words:**Systematic review, xerostomia, clinical trial, hyposalivation.

## Introduction

**Figure 1:**
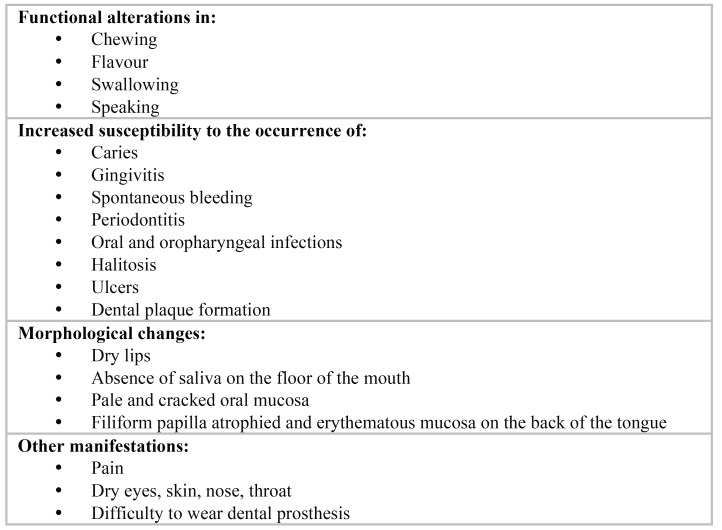
Signs and symptoms of hyposalivation in the oral cavity.

The total or partial loss of saliva causes serious oral consequences, manifesting as an uncomfortable feeling of dry mouth (xerostomia) and presenting numerous signs and symptoms mainly in the mucous membranes, lips, tongue, salivary glands and teeth (Fig. 1) (1,2). In the majority of cases, dry mouth is caused by hypofunction of the salivary glands, acute or chronic, with or without xerostomia. In other cases, xerostomia is not accompanied by an actual loss of saliva (3). In older people, the most common cause is the use of medications with potential xerostomic effects, mainly anticholinergic, sympathomimetic, sedative-hypnotics, opiates, antihistamines and muscle relaxants (4). In other situations, a dry mouth is mainly due to the radiation received by patients with cancer in the head / neck area or in patients with certain autoimmune diseases such as Sjögren’s Syndrome.

Despite the different etiological origins of dry mouth, the available treatment does not differ from one situation to another, except in cases of drug-induced xerostomia, in which case, the therapeutic strategy is to terminate the drug causing the sensation of dryness or substitute it for another with less xerostomic effect (5). So far there is no clear and effective treatment for every case, with different results and little scientific evidence in some cases, outlined in the latest systematic reviews published in 2011 (6) and 2013 (7) through the Cochrane Collaboration.

The aim of this study was to systematically review the latest available evidence regarding the treatment of dry mouth, regardless of the cause of the problem.

## Material and Methods

For this review, we have followed PRISMA and the Cochrane Collaboration criteria (8,9) in terms of the formulation of review questions, construction of the search strategy; definition of inclusion and exclusion criteria; location and study selection; assessment of the quality of work; data extraction and interpretation. After development of the work protocol, the following review question was posed: “In older subjects with xerostomia and/or hyposalivation, regardless of etiology, what pharmacological or non-pharmacological treatments have positive results in terms of decreasing symptoms or increasing salivary flow?”

The population included in the review were “older” subjects, complaining of dry mouth either from the consumption of possibly xerostomic drugs, or cases of patients diagnosed with Sjögren’s syndrome or other systemic diseases or those patients who have received radiation for head and neck cancer. Using the filters available in PubMed, the search was limited to “Clinical Trials”, from 2006 to March 2015, in English, only in humans and using base data from MEDLINE. MeSH terms and the general search strategy were based on the following: (xerostomia OR Dry Mouth Syndrome) AND (OR Elderly Aged). After an initial selection of studies and after reviewing the title and abstract, the following were discarded; those that were not really clinical trials (although these would have been included as such by the computer system), literature reviews, systematic reviews and letters to the Director. Subsequently, a new filter was applied to the selected items to assess the methodological quality. To do this we used the “Oxford Quality Scale” tool that gives scores between 0-5 depending on the testing method of randomization, blind testing and loss assessment (10). The studies have been divided into three categories: 1) clinical trials that tested pharmacological treatments like pilocarpine or cevimeline; 2) clinical trials that tested other non-pharmacological or artificial saliva products; 3) clinical trials that tested alternative treatments like acupuncture or electro-stimulation.

## Results

**Figure 2:**
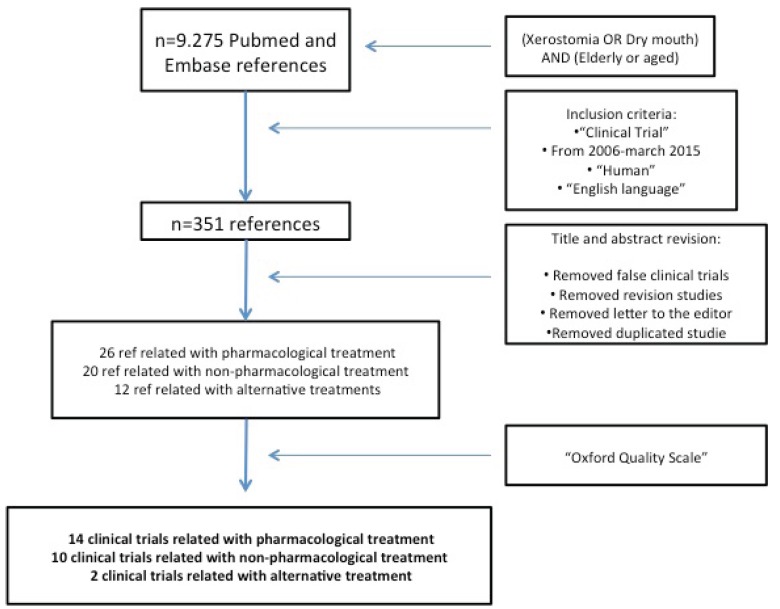
PRISMA flow chart to demonstrate the methodology applied to selected articles.

In the initial search of the Medline database, a total of 9,275 references were obtained (Fig. 2). After the first filter, a total of 351 papers were obtained. During the review of the titles and abstracts of these papers the following were discarded: those that were not really designed as a clinical trial; review papers; letters to the editor and any duplicates. After classification by type of treatment that was being tested, 26 studies evaluating various drug treatments were identified, another 20 were testing other non-pharmacological commercial products or artificial saliva products and 12 were testing alternative treatments. Finally, the 58 selected works were analysed in depth and after applying the scale proposed by Jadad *et al.* (Oxford Quality Scale) (10), a total of 26 well-designed clinical trials with score 4 or 5 were identified ( Table 1 and  Table 1Continue -  Table 3). The final 26 studies were reassessed in duplicate by two investigators, agreeing the score received where there was any discrepancy between the two. Few studies showed how the sample size was calculated, with the selected populations being between 20 and 570 individuals.

**Table 1:**
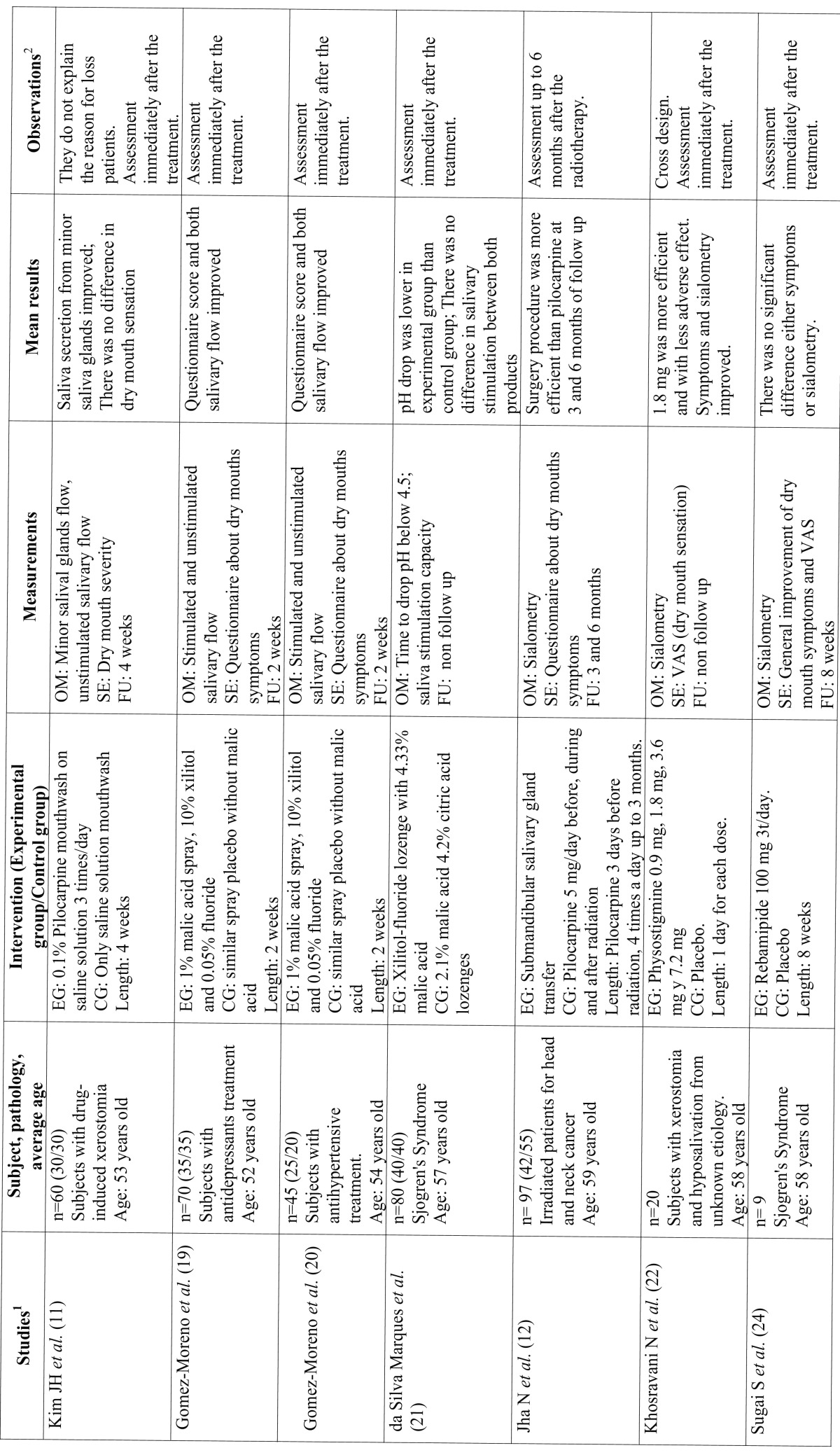
Studies related to pharmacological treatments.

**Table 1 Continue:**
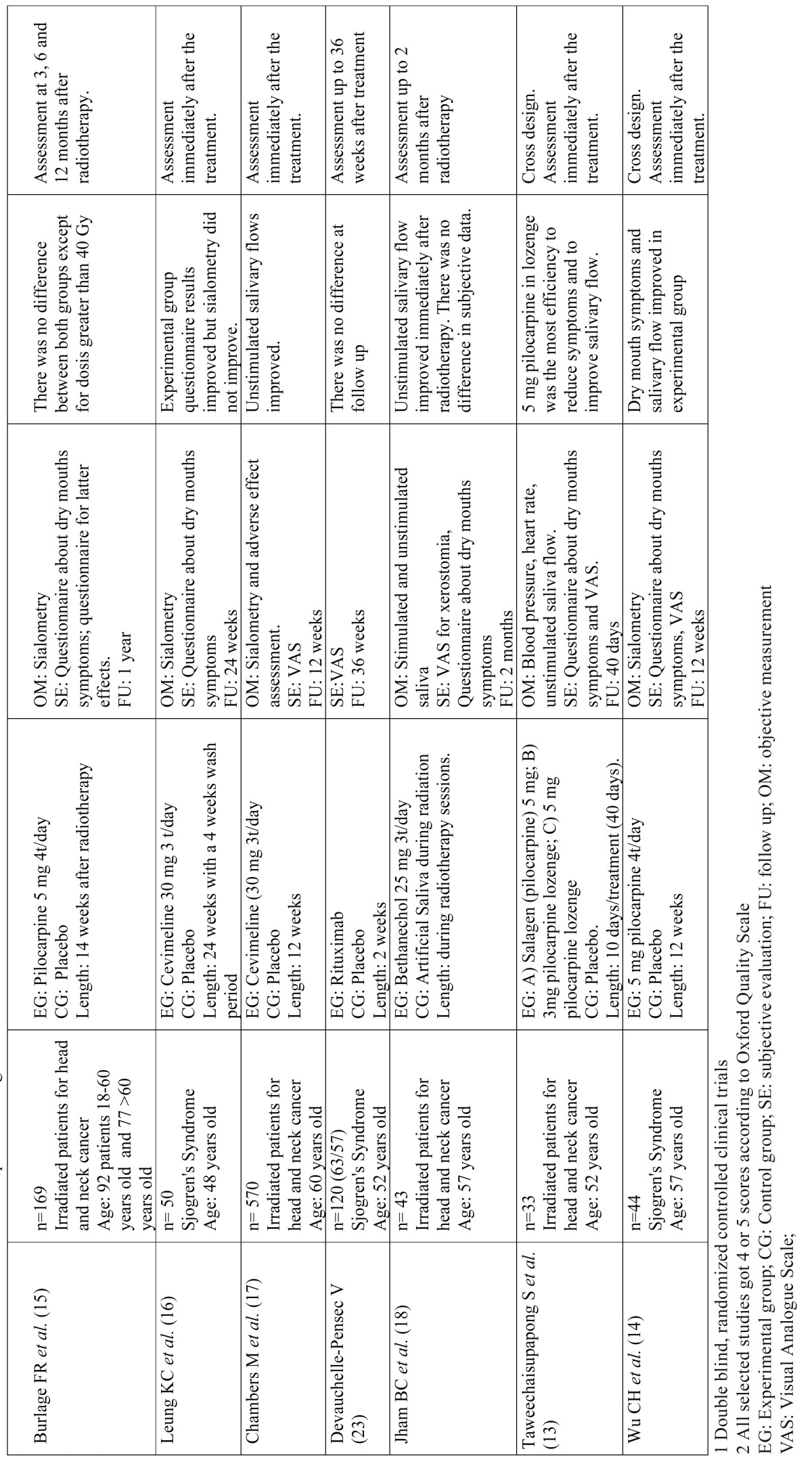
Studies related to pharmacological treatments.

**Table 2:**
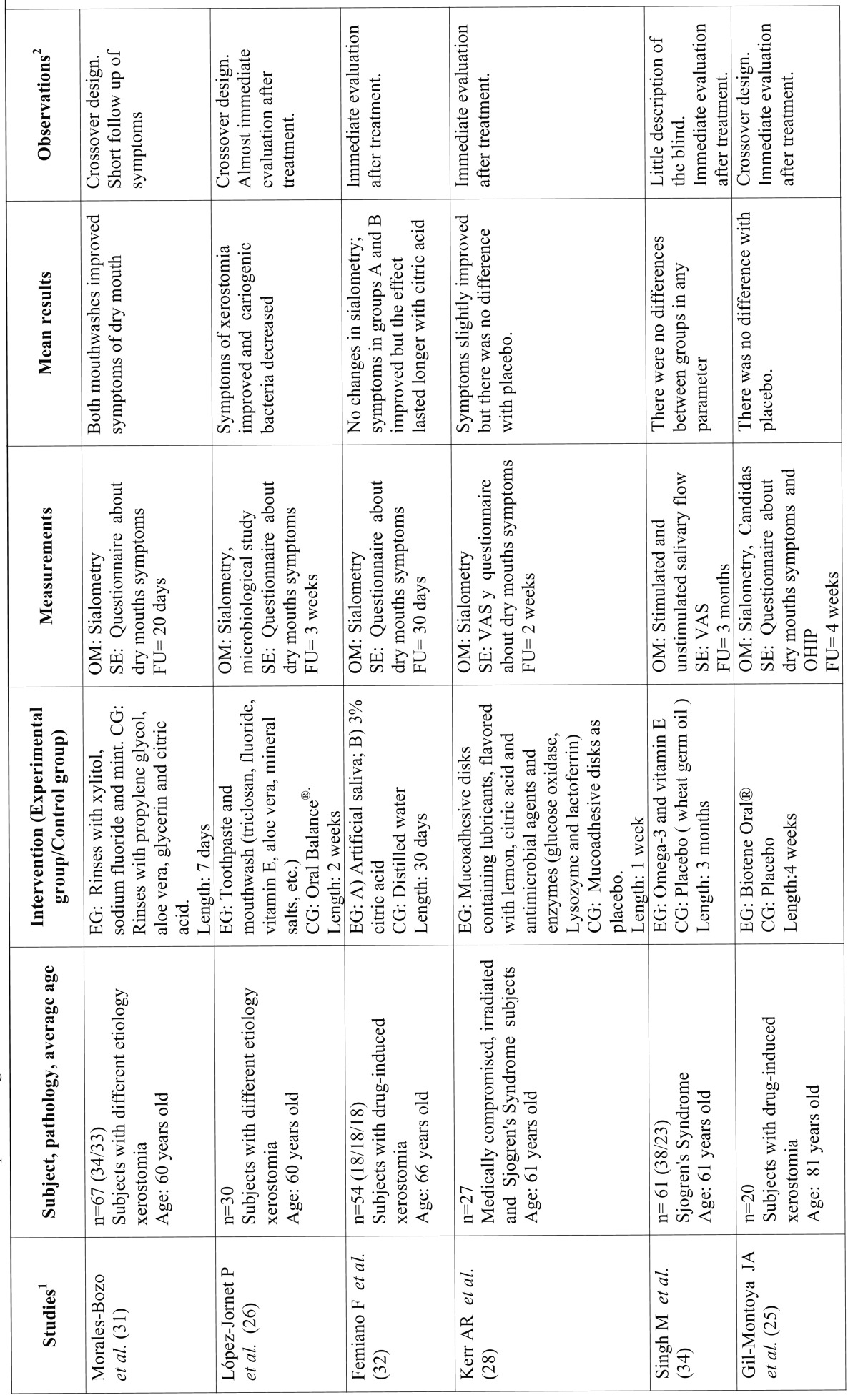
Studies related to non-pharmacological treatments.

**Table 2 Continue:**
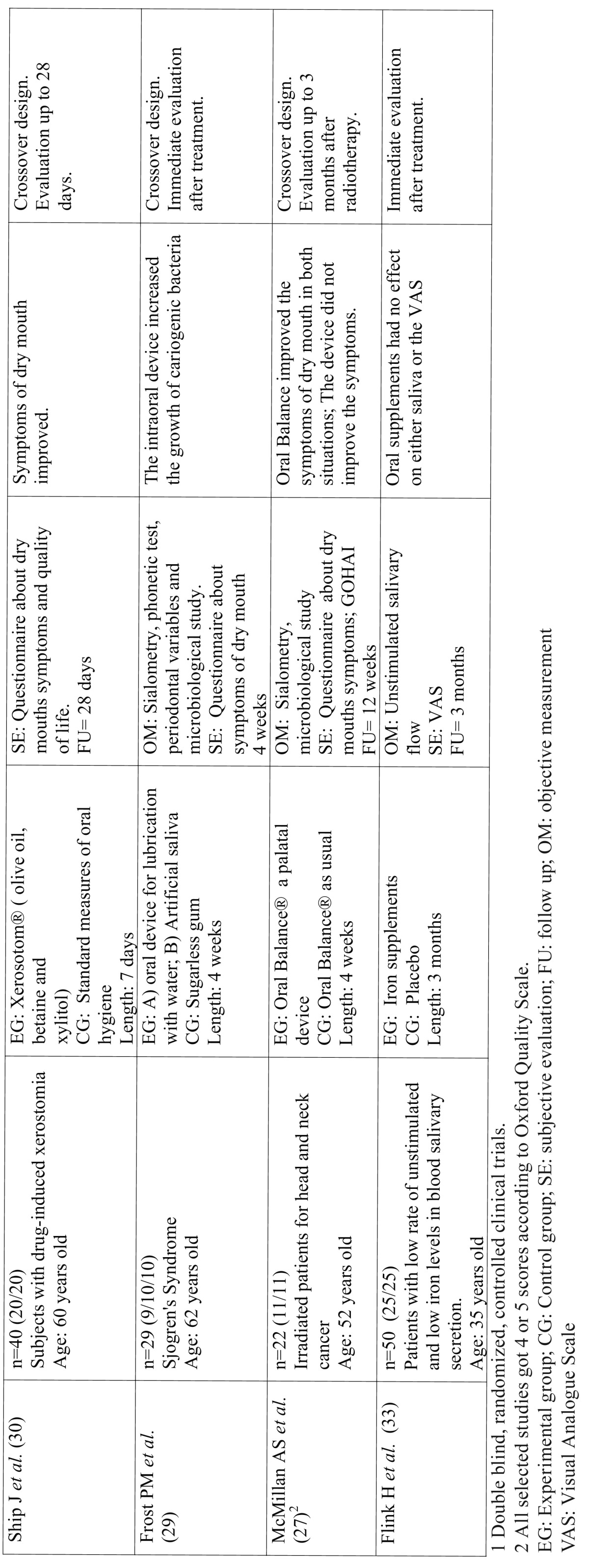
Studies related to non-pharmacological treatments.

**Table 3:**
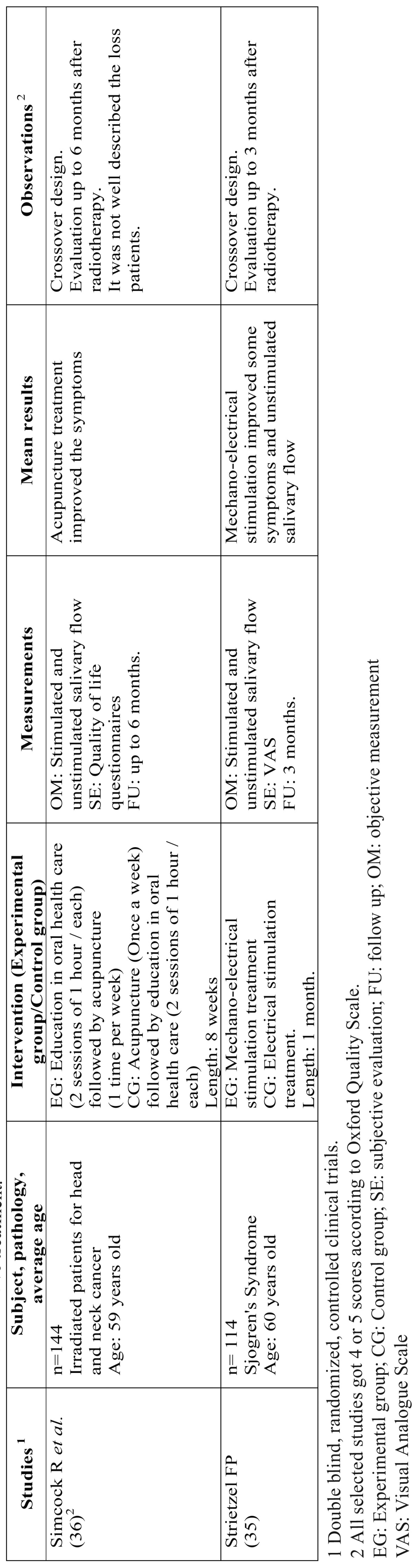
Studies related to alternative treatment.

- Trials related to drug treatment.

A total of 14 clinical trials were identified, a priori properly randomized, double-blind, where there were given reasons for the possible loss of subjects after the test (except for Kim JH *et al.* (11) ( Table 1 and  Table 1 Continue ). Therefore, these selected trials obtained the highest score according to the Oxford Quality Scale. The remaining studies were not selected mainly because they were not designed as clinical trials, were not well controlled or the authors gave no information either on blind testing or the randomisation processes, so the risk of bias was considerably increased. Among the selected studies, as many crossover trials were found in the experimental group as in the trials with two or three parallel groups. Most were testing cholinergic agents (muscarine agonists) such as pilocarpine (11-15) cevimeline (16,17) and bethanechol (18) and the rest checked the efficacy of drugs or agents such as malic acid (19-21) or physostigmine (22). Finally, two studies analysed the improvement of symptoms of dry mouth in patients with pathologies such as Sjogren’s Syndrome, acting directly on the pathophysiology of the disease, with specific monoclonal antibodies such as Rituximab (23) or Rebamipide (24), drugs derived from fluoroquinolone with a protective action for gastric mucosa among others. Three of the selected trials evaluated the action of the intervention months after the application of the drug treatment. The rest were carried out only to the end of the intervention, with no follow-up.

Five of the trials published at this time focused on subjects irradiated for head and neck cancer (12-15,17,18). In these cases, despite obtaining improved on stimulated and unstimulated salivary flow, not all had clearly improved symptoms of dry mouth. The dose of 5 mg pilocarpine four times daily was the most common and most effective, especially when the tablets were allowed to dissolve in the mouth, showing no significant adverse effects of this treatment. The way of administering the drug during or after radiation and the different doses made it difficult to compare or draw general conclusions from these studies. The study of Burlage *et al.* with pilocarpine versus a placebo has been revised with longer follow-up and with a larger sample size (15). The use of cevimeline (16,17) and bethanechol (18) also had positive results in salivary flow as did the surgical transposition of the submandibular gland compared with treatment with pilocarpine (12). The only selected study where biological therapy with rituximab was used also had a large sample size and 3 months follow-up (23).

Of the five works selected and carried out on patients with Sjögren’s Syndrome (14,16,21,23,24), only that published by CH Wu *et al.* (14) showed positive results both in symptoms and in sialometry, in this case testing pilocarpine in doses of 5 mg, four times daily. The other studies, testing with rituximab (23), malic acid (21), rebamipide (24) or cevimeline (16) showed no clear significant changes which would warrant or support the idea of using these drugs to treat the underlying disease or its consequences. Only in the study published by KC Leung *et al.* (16), were the symptoms were slightly better in the experimental group though sialometry was not.

Finally, in published trials on people with drug-induced xerostomia (11,19,20) or unknown causes (22), the results were mixed. In the study of Gómez-Moreno *et al.* on patients taking antidepressants (20) and antihypertensive (19), using a spray of 1% malic acid improved both symptoms of dry mouth and salivary flow. The effect was also seen when the drug Physostigmine as a parasympathometic was used at doses of 1.8 mg / day and without significant adverse effects (22).

- Trials related to non-pharmacological treatment.

The studies that were not included in this section presented a low level of evidence through inadequate design. Where these studies were of a crossover design, they were excluded because many were not randomized; inclusion criteria was not mentioned or was very vague; there was no explanation about dropouts or losses or they were not double-blind. In other cases, the sample size was particularly low. Finally, eight successfully designed studies were identified, using artificial saliva or salivary substitutes in patients with xerostomia and two where the efficacy of oral supplementation for improving the signs and symptoms of dry mouth (Table 2 and 2 Continue) were tested.

In four of the studies, an assessment of the effect of Biotene / Oral Balance®, an enzymatic system with an antimicrobial effect based on glucose oxidase, lactoperoxidase, lysozyme and lactoferrin glucose, was made. In the study of Gil Montoya *et al.* (25) it was tested against a placebo, evaluating dry mouth symptoms and showing no differences between the two groups. In the study by López-Jornet *et al.* (26) it is compared with a mouthwash with triclosan (antiseptic) and remineralising ingredients, such as fluorides and mineral salts. An improvement of symptoms was found with the triclosan mouthwash and also a significant decrease of cariogenic bacteria. Mc Millan *et al.* (27) compared the effect of Biotene / Oral Balance® against the efficacy of an intraoral appliance to maintain lubrication of the oral mucosa. A significant improvement was observed in the symptoms of dry mouth in the Oral Balance group whilst patients with the intraoral device reported severe discomfort. Finally, in the study by Kerr *et al.* (28) where muco-adhesive discs with lubricants and antimicrobial agents (glucose oxidase, lysozyme and lactoferrin) were tested versus placebo discs, slight improvements were noted but without significant differences between the two groups.

Frost *et al.* (29) had already tried to maintain oral lubrication with water or artificial saliva by means of an intraoral device and compared this with a group who chewed sugarless gum. They concluded that the oral device produced difficulties on speech and cariogenic growth of germs. In the study by Ship *et al.* (30) a crossover study was carried out on 40 adult patients taking multiple drugs with xerostomic effects (2 groups of 20 subjects) where the patients were given a mouthwash based on olive oil, betaine and xylitol. A quantitative sialometry was performed and some increase in salivary flow rates were observed when using the product which reduced discomfort. There were no significant adverse effects.

Morales-Bozo *et al.* (31) tested with a mouthrinse containing xylitol, sodium fluoride, cetylpyridinium and mint over another with propylene glycol, aloe vera, glycerin, and citric acid. Both mouthwashes improved the symptoms of dry mouth. Femiano *et al.* (32) in their study compared three groups of water-based artificial saliva, one containing cellulose, sorbitol and mineral salts, another with 3% citric acid and the last group with water. Changes were not observed in the rates of unstimulated salivary flow. However, there was an improvement in the symptoms of the group with artificial saliva and citric acid, the latter being where the effects were longer lasting.

Finally, in connection with oral supplements in the study by Flink *et al.* (33), the effect of iron supplements (oral) on unstimulated salivary flow rate versus a placebo was investigated in patients diagnosed with hyposalivation and low iron. These supplements had no significant increase in the subjects’ secretion of unstimulated saliva. Likewise, other authors (34) compared Omega-3 and vitamin E supplements against wheat germ oil in Sjögren’s Syndrome patients. Both rates increased salivary secretion (stimulated and unstimulated) without obtaining higher significant benefits in the supplements with Omega 3 and vitamin E.

- Trials related to alternative treatment.

Taking the criteria for inclusion into consideration, only two studies were selected in this review within the selected dates ( Table 1). The most common reason for exclusion of the remaining studies was that the study design was not compatible with a clinical trial.

Strietzel *et al.* (35) examined the efficacy of intraoral electrostimulation in symptoms and signs of xerostomia in patients with this condition. This technique showed a significant improvement in the short term (3 months) in symptoms such as frequency and severity of dryness and difficulty swallowing and in the long-term (5 months) in symptoms such as frequency and severity of dryness, oral discomfort, difficulty in sleeping and talking and the rate of unstimulated salivary secretion.

Other authors such as Simcock *et al.* (36) examined the efficacy of acupuncture compared with the delivery of two sessions of oral education (advice on diet products to relieve xerostomia and oral hygiene) in patients treated with radiotherapy. After 8 weeks of application, the patients treated in this group had a significant improvement in symptoms (dry mouth, sticky saliva, need to drink to swallow food and getting up at night needing a drink). There were no differences in rates of stimulated or unstimulated salivary secretion.

## Discussion

According to the results of the trials included in this review, the efficacy of different therapeutic strategies for the control of symptoms and signs derived from hyposalivation, regardless of their origin, are still not strong enough to recommend a particular treatment, either pharmacological or not. Most treatments tested and used in patients with xerostomia temporarily improve symptoms and, to some extent, salivary flow, but without medium or long term control in all cases, making the use of such therapeutical strategies difficult and unpredictable. In general, the sample sizes of the studied clinical trials were small or moderate and short-lived, especially the non-pharmacological treatment trials and alternative treatments, which also call for solid and consolidated results. Ten of the 26 selected studies were crossover trials, a design that allows more efficiency in determining patient preferences.

When hyposalivation and its oral consequences comes from irradiation of the parenchyma, pilocarpine is the most-used parasympathomimetic drug with the best results, but always where there is some residual function of the parenchyma. Studies were carried out using different doses (3 and 5 mg), different times of use (during or after radiotherapy) and different dispensing means (swallowing or dissolving tablets in the mouth or rinses). Localised treatment with pilocarpine by dissolving tablets (13) or rinses to 0.1% seems more effective than systemic administration (11), although further studies to strengthen the scientific evidence and to determine the necessary dose of topical pilocarpine in order to really improve the effectiveness of this system. Studies with a longer follow-up to determine long-term effects were conducted with pilocarpine, such as the work published by Burlage *et al.* (15) where it was tested against a placebo or where Jha *et al.* compared it with the surgical transposition technique (12). Trials with other drugs such as bethanechol and cevimeline have also been successful in improving the levels of salivary flow and symptoms in this type of patient (16,18). However, they have not been tested against pilocarpine so it is not known whether they improve the cost / benefit ratio. None of the studies in this review observed the systemic adverse effects associated with pilocarpine or other parasympathomimetic drugs, such as sweating, nausea, fever or diarrhoea. This is probably due to the short follow-up period of the studies reviewed (37), except for that of Jham *et al.* (18) where patients were reviewed for two months after completion of radiotherapy.

In the case of Sjögren’s Syndrome there has also been a lack of important developments in recent years in terms of drug treatment. Pilocarpine again remains the only drug used in trials showing clear improvements in salivary flow and symptoms (14). Trials with rebamipide and malic acid showed no significant differences from their placebos. Only in the work published by Leung *et al.* (16) was an improvement of symptoms observed in the experimental group, though not in the sialometry. This fact, according to some authors, is due to the effect of parasympathomimetic drugs on the minor salivary glands, rather than on the parenchyma of the major salivary glands, causing a major lubrication of the oral mucosa without resulting in a real increase in the total amount of saliva secreted (13). Emerging biological therapies in recent years have not so far produced anticipated results for Sjögren’s Syndrome. Specifically, rituximab, a monoclonal antibody with proven activity in diseases such as lymphoma or rheumatoid arthritis, has not always proven to be more effective than the placebo itself (23) without knowing in which patients this and other biological therapies obtained good results (38). However, this latter was the only trial that followed patients for 36 months to identify the results of long-term intervention.

Few studies focused on the pharmacological treatment of xerostomia where the cause is due to the consumption of drugs, in addition to not knowing what would happen in the long-term and to medium sample sizes. The use of malic acid with fluoride and xylitol, both spray and tablets, have been successful in terms of symptoms and sialometry (19-21). However, more tests are needed with larger sample sizes and where products are applied for a longer period to assess the possible adverse effects of acid on tooth enamel. A new approach from Khosravani *et al.* (22) assessed the capacity of physostigmine (a plant-derived alkaloid acetylcholinesterase inhibitor) as a sialogogue drug in patients with xerostomia of unknown cause (patients not suffering any disease that would justify it like Sjögren , sarcoidosis, etc), or who had received radiation and who had not been labelled a priori as taking xerostomic drugs. In these cases (20 subjects), a clear improvement was observed in symptoms and salivary volume secreted in the following 2-3 hours after application of a solution of physostigmine.

In all the studies analysed where a mouthwash was used, the symptoms improved even after using water or a placebo (25-27,30,31). However, no short to medium term monitoring was carried out so the durability of the treatment applied is unknown. It seems logical that in these cases the treatments applied should continue as long as the etiology exists (xerostomic drugs, autoimmune syndromes, etc). This shows that single lubrication of the oral mucosa is positive with respect to xerostomia. It highlights the study of Morales-Bozo, which has an acceptable sample size, a crossover design but a short follow-up after the application of a mouthwash. In both working groups the results were very positive (31). The use of intraoral devices such as a deposit has not been proven useful (29). The problem is that they do not last long enough to produce an improvement in the quality of life of these patients. However, the effect of using citric acid in a salivary substitute does seem to provide increased and improved comfort for somewhat longer periods (31,32). These salivary substitutes are often used when the xerostomia has been caused by the irreversible destruction of the salivary hyposialia by parenchyma or because sialagogues can not be administered to patients because of the adverse effects (26). Lubricants and salivary substitutes are only a useful palliative treatment when they are administered continuously.

The drawbacks shown with intraoral devices for storing artificial saliva has also been a trend in the studies reviewed here. These devices produce inconvenience and discomfort for patients and could even alter the oral environment favouring the growth of cariogenic bacteria. Conversely, use of a mouthwash with triclosan had the opposite effect by decreasing such microorganisms. In an interesting study carried out some time prior to our review (39), the application of a reservoir of artificial saliva seemed to be a good therapeutic alternative for improving the symptoms of xerostomia because, although no significant differences were found in the total score in the questionnaire assessing quality of life, the reservoir in itself reduced the number of impacts affecting the daily life of the patient. The failure to find corresponding differences in the total score could be due to the short follow-up period of the study (1 month) since the modifications made in the questionnaire and application form affect the mathematical properties of the original questionnaire.

The similarity in oral symptoms presented by patients with iron deficiency and in patients with xerostomia and / or hyposalivation and the known role of iron in cellular metabolism may make iron administration a therapeutic option for improving hyposalivation (40). However, it has been shown that this treatment is not effective. Oral supplementation with Omega-3 and vitamin E for patients with Sjögren’s Syndrome, however, could be beneficial in improving salivary secretion rates (stimulated and unstimulated) (34). The result of such supplements were not significantly better than supplements with wheat germ oil though the reason for this may lie in the fact that the latter also had small amounts of Omega 3 and vitamin E (34).

The use of alternative stimulating agents could be a good therapeutic option for the treatment of xerostomia / hyposalivation because, in most cases, these have no side effects or those that have been described are limited. Among these alternatives, the oral electrostimulation and acupuncture have shown to be effective treatments to improve certain symptoms affecting patients with xerostomia (35,36). Oral electrostimulation had the added advantage that, even in patients who had a higher therapeutic challenge, being completely devoid of salivary secretion capacity at baseline, showed improvement in the rate of unstimulated salivary secretion. The two studies selected for this review had acceptable sample sizes and follow-ups of six and three months after completion of the intervention, reinforcing the results.

The inherent limitations of systematic reviews in general are also evident in our work. Despite performing an initial broad search strategy, with subsequent limitations, some tests may not have been identified. For example, those which do not describe their population as “elderly” or those written in languages other than English. We recognize that these issues may incur potential bias for this review. Similarly, some studies in implementing the “Oxford Quality Scale” excessively or inadequately, may have been incorrectly selected or not, as a result of the publication description made by the author.

In short, as evidenced by the data found in the selected publications in this review, pilocarpine, the sialogogue drug, has produced the best results in patients with xerostomia due to radiation in head and neck cancer or diseases such as Sjogren’s Syndrome. In patients with purely drug-related dry mouth there is some positive evidence in the use of malic acid along with other elements that counteract the harmful effects on dental enamel. In general, the lubrication of the oral mucosa reduces symptoms, although the effects are short-lived so far. It is necessary to conduct further trials, preferably crossover in design, with large sample sizes and long-term monitoring of the effects of the intervention.
